# Supplementary material for: Genome-Wide Circular RNA Expression Patterns Reflect Resistance to Immunomodulatory Drugs in Multiple Myeloma Cells
Source: Cancers (Basel). 2021 Jan 20;13(3):365. doi: 10.3390/cancers13030365 (PMC7930955; doi:10.3390/cancers13030365)
Supplement: Supplementary file 1 [file cancers-13-00365-s001.zip › cancers-1044145-supplementary.pdf]

cancers

MDPI

Supplementary materials: Genome-Wide Circular RNA Expression Patterns Reflect Resistance to Immunomodulatory Drugs in Multiple Myeloma Cells

Theresa Jakobsen, Mette Dahl, Konstantinos Dimopoulos, Kirsten Grønbæk, Jørgen Kjems and Lasse Sommer Kristensen

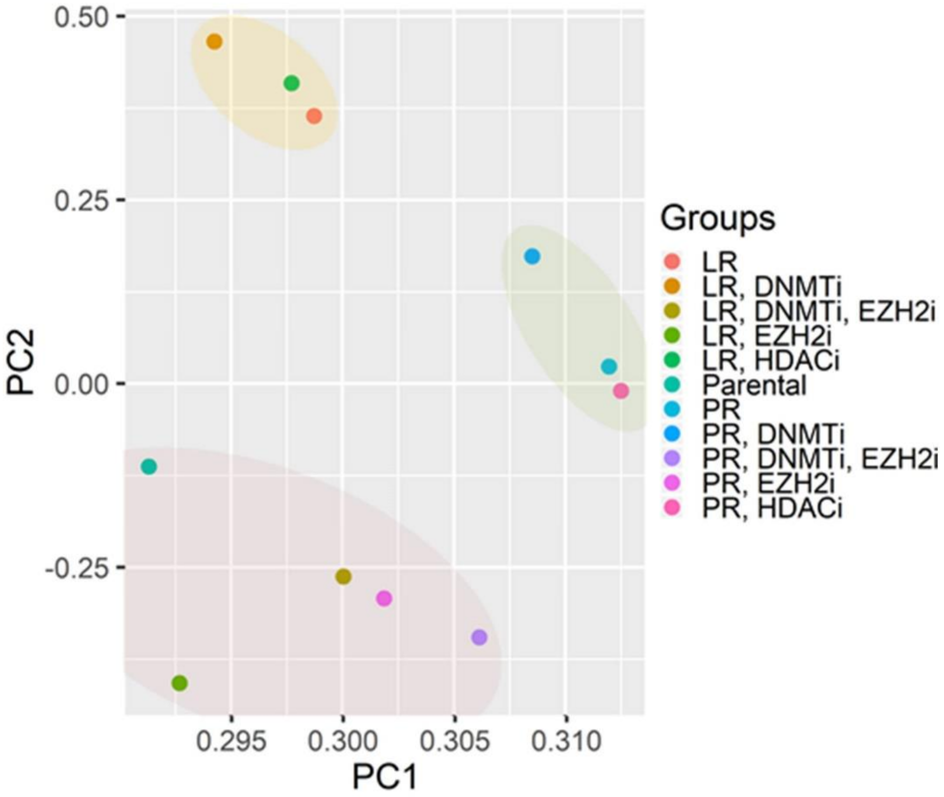

Figure S1. Principal component analysis (PCA) of all 6,368 circRNAs detected in the 11 samples (analyzed by RNA-Seq).

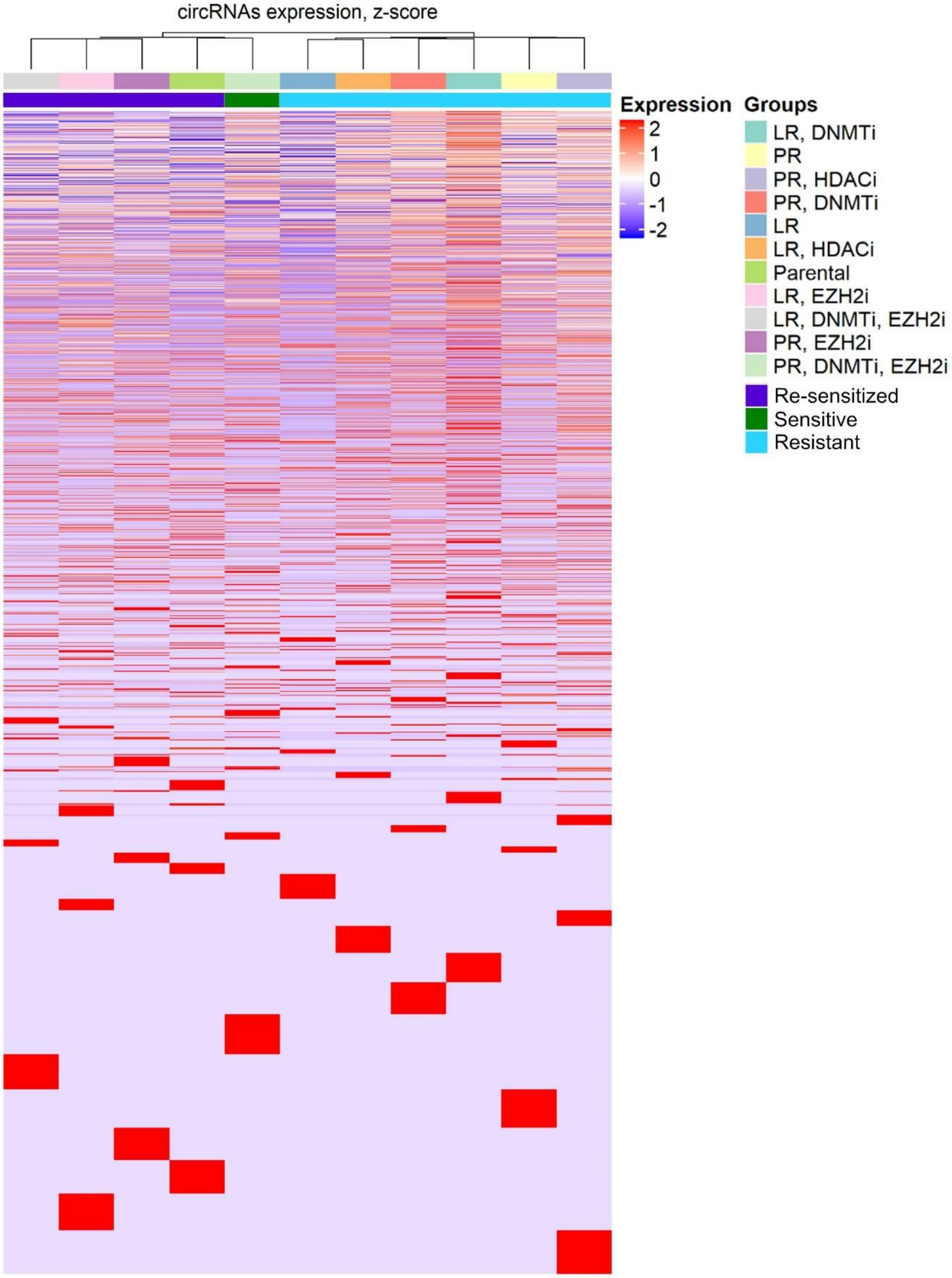

Figure S2. Heatmap showing expression and unsupervised clustering of all 6,368 circRNAs detected in the 11 samples (analyzed by RNA-Seq).

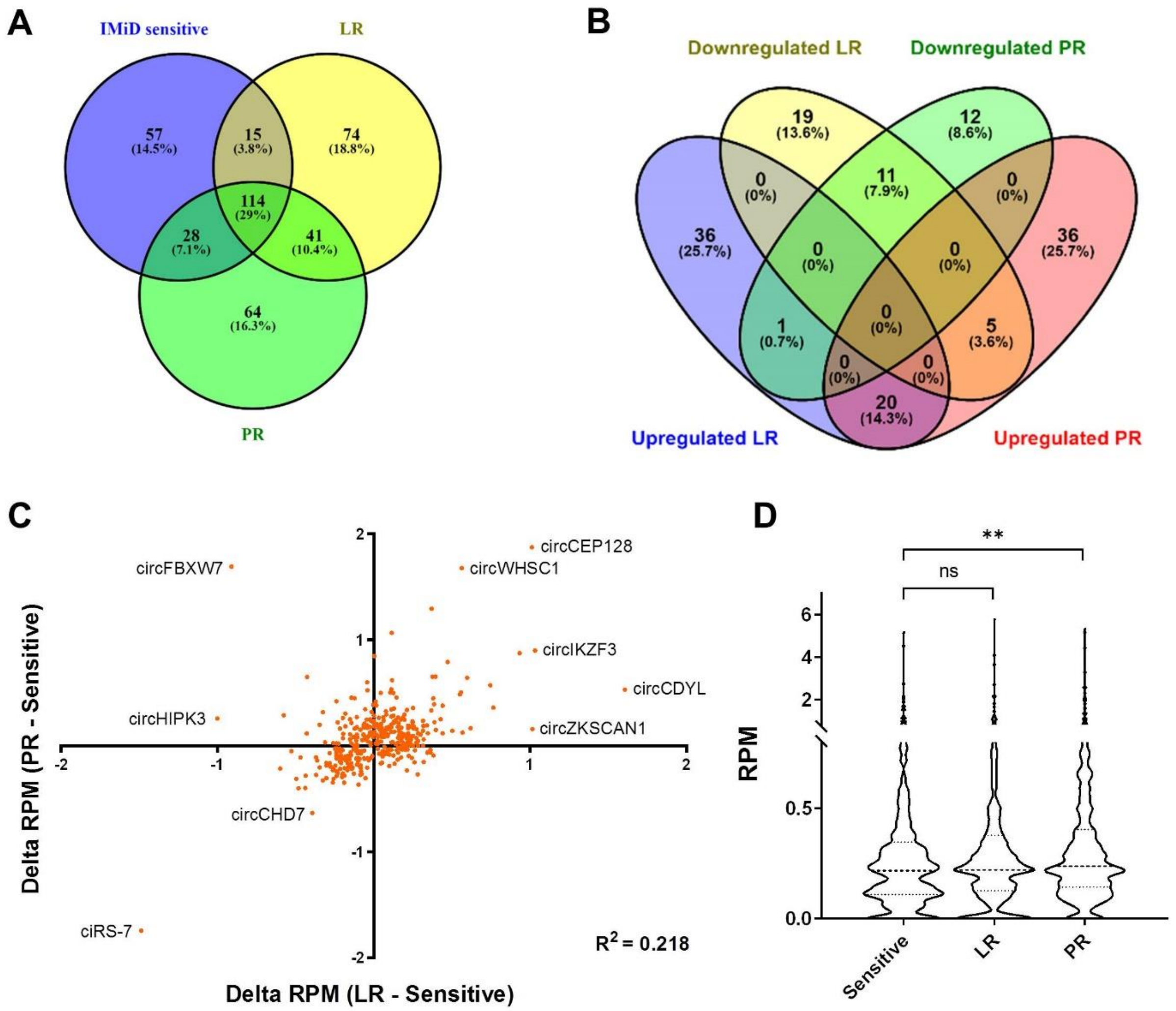

Figure S3. Comparison of circRNA expression changes in LR vs PR cells. **(A)** Venn diagram illustrating the overlap of high abundance circRNAs (with RPM>0.2) detected in parental (IMiD sensitive), LR and PR cells. **(B)** Venn diagram illustrating the overlap between up- and downregulated circRNAs in LR and PR. **(C)** Scatter plot showing the up- and downregulated circRNAs in LR and PR plotted against each other, with corresponding R^2 value. **(D)** Violin plot showing all circRNAs expressed in parental (sensitive), LR and PR cells, in RPM, **$p<0.01$ (Mann-Whitney test). LR, lenalidomide-resistant, PR, pomalidomide-resistant.

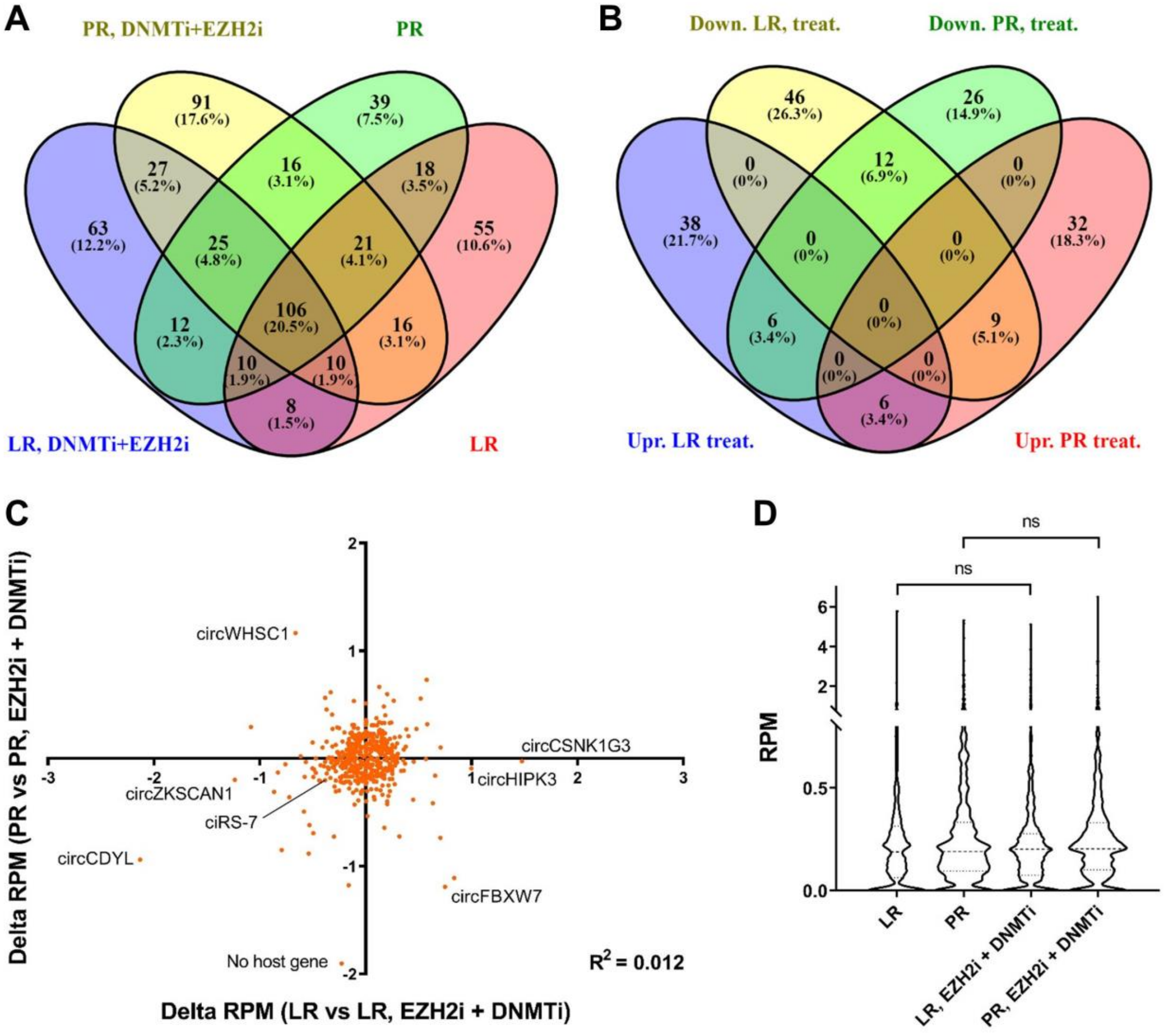

Figure S4. CircRNA expression changes upon combined DNMT and EZH2 inhibition. **(A+B)** Venn diagrams illustrating the overlap between circRNAs expressed in LR, LR–treat, PR and PR–treat **(A)** and the up- and downregulated circRNAs in LR-treat and PR-treat **(B)**. **(C)** Scatter plot showing the up- and downregulated circRNAs in LR-treat and PR-treat plotted against each other. **(D)** Violin plot showing the overall expression of circRNAs in RPM. LR, lenalidomide-resistant, PR, pomalidomide-resistant, Down., downregulated, Upr., upregulated, LR-treat, LR cells treated with EZH2i and DNMTi. PR-treat, PR cells treated with EZH2i and DNMTi.

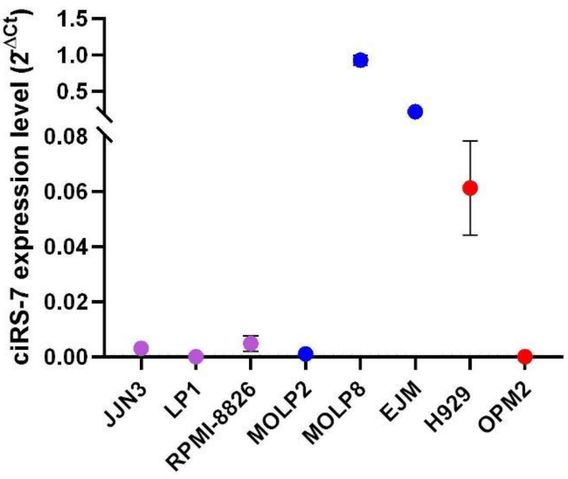

Figure S5. ciRS-7 expression in eight MM cell lines (purple=primary resistant cell lines, blue=partially resistant cell lines, red=sensitive cell lines). Relative ciRS-7 expression (RT-qPCR), error bars reflect technical triplicates.

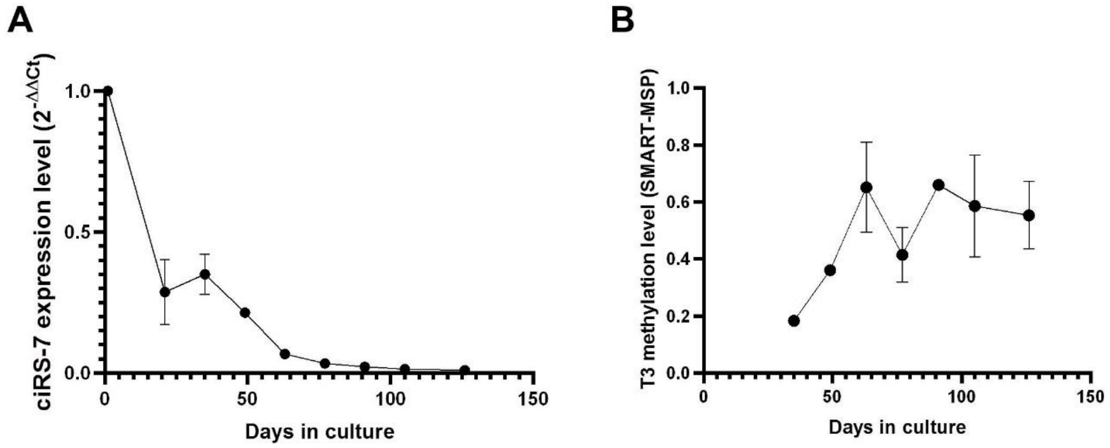

Figure S6. Loss of ciRS-7 expression over time in parental cells. **(A)** Relative ciRS-7 expression (RT-qPCR) of NCI-H929 cells growing in culture. Error bars reflect technical triplicates. **(B)** Relative methylation level (SMART-MSP) of T3 in NCI-H929 cells growing in culture. Error bars reflect technical triplicates.

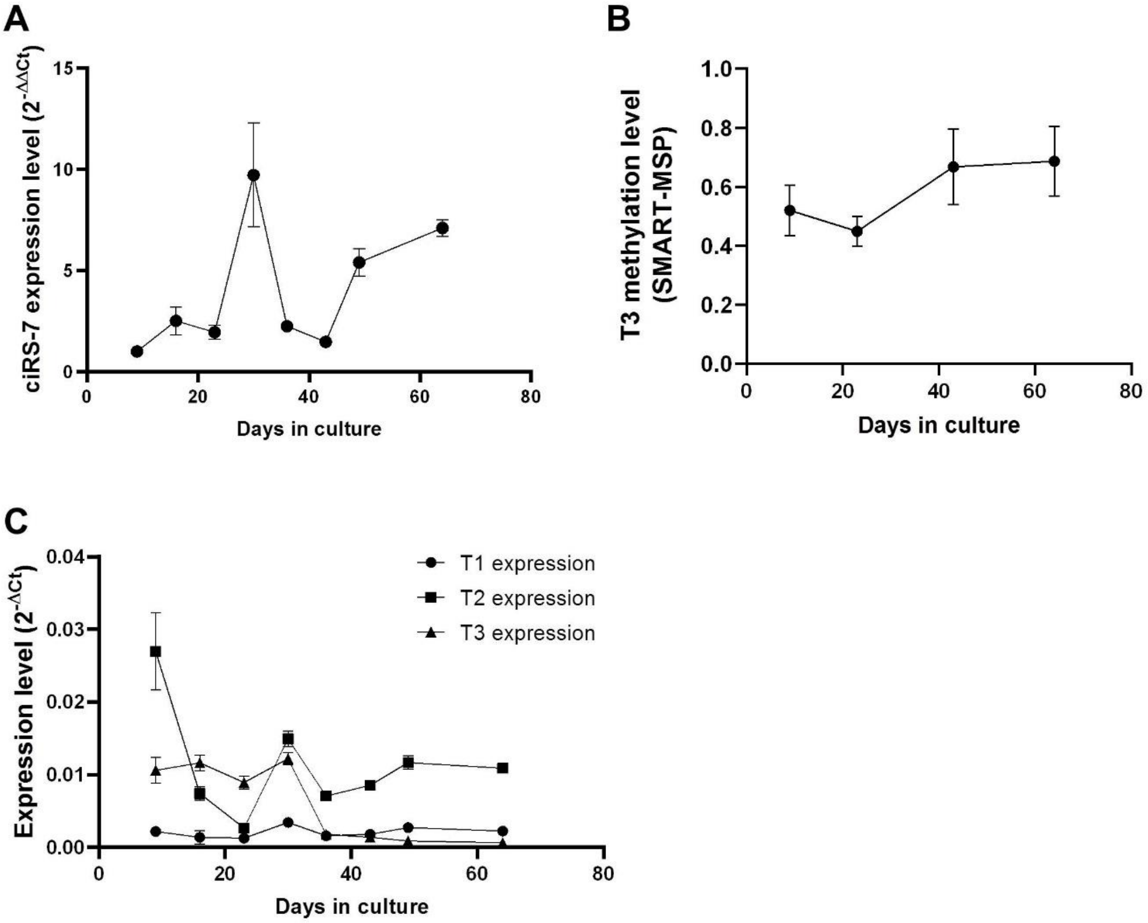

Figure S7. ciRS-7 expression over time in MOLP8 cells. **(A)** ciRS-7 expression (analyzed by RT-qPCR) in MOLP8 cells growing in culture. Error bars reflect technical triplicates. **(B)** T1, T2 and T3 expression (analyzed by RT-qPCR) in MOLP8 cells growing in culture. Error bars reflect technical triplicates. **(C)** Relative methylation level (SMART-MSP) of T3 in MOLP8 cells growing in culture. Error bars reflect technical triplicates.

Table S1. sisiRNA and siRNA sequences used for ciRS-7 knockdown.

Target	Sequence
ciRS-7 back-splice junction	AAAACCCUGGAUAUUGCAGAC
ciRS-7 internal sequence	UUGGAAGACACAAGUAGGCGCU [1]
circIKZF3 back-splice junction 1	UCCGCACUUGCAAUGAAUUUC[dT][dT]
circIKZF3 back-splice junction 2	GCACUUGCAAUGAAUUUCUGA[dT][dT]

[1] The underlined nucleotides are locked nucleic acids (LNAs) that increase the hybridization properties of the sisiRNA.

Table S2. Primers for RT-qPCR.

Target gene/transcript	Forward primer	Reverse primer
LINC00632 (T1)	5′GACATGGAATTCCTGGATCA	5′GTCACTAGGTGGCAAACGTG
LINC00632 (T2)	5′ CCGTCCCTTGCTGATTGTAT	5′ GTCACTAGGTGGCAAACGTG
LINC00632 (T3)	5′GAGGCGGTTAAGGAGAGGAG	5′TCAGGAGTCAAGGTCAGGCTA
ciRS-7	5′ACGTCTCCAGTGTGCTGA	5′CTTGACACAGGTGCCATC
Linear IKZF3	5′ ACATTTCTTCAGAGCACTGACC	5′ TCTGCTTTGATGTGTCTTGCCT
Circular IKZF3	5′GAAAAAGCTCAATGCCTCAGAAATTC	5′ TCTGCTTTGATGTGTCTTGCCT
SF3A1	5′TCCATCCGTGAGAAGCAGAGC	5′TCTGGATCTCCTCCTCACCG
PUM1	5′CATGCCAGGTTATCCGGTGT	5′GCGCCTGCATTCACTACAAG

Table S3. Methylation-specific primers used for SMART-MSP.

Target gene/transcript	Forward primer	Reverse primer
LINC00632 (T3)	5′ gtgg**cg**agtTaggagT**cg**ac [1]	5′ tctAAaAaAacc**cg**ccctA**cg** [1]
Alu control	5′ GGTTAGGTATAGTG GTTTATATTTGTAATTTTAGTA	5′ ATTAACTAAACTAATCTTA AACTCCTAACCTCA

[1] For the LINC00632 (T3) primer set, upper-case T indicates that the base was C before sodium bisulfite conversion and CG sites are indicated in bold.

Table S4. Methylation-independent primers for amplification of bisulfite-treated DNA and subsequent Sanger sequencing.

Target gene/transcript	Forward primer	Reverse primer
LINC00632 (T3)	5′ggTataTtgTaaTtaggtTagaagtgaT [1]	5′ gagtTTtTTTTagagaggggagg [1]

[1] Upper-case T indicates that the base was C before sodium bisulfite conversion.
